# Prognostic value of the triglyceride-glucose index for adverse cardiovascular outcomes in young adult hypertension

**DOI:** 10.1186/s40885-024-00274-9

**Published:** 2024-09-01

**Authors:** Chen Li, Yu Zhang, Xueyi Wu, Kai Liu, Wei Wang, Ying Qin, Wenjun Ma, Huimin Zhang, Jizheng Wang, Yubao Zou, Lei Song

**Affiliations:** 1 Department of Cardiomyopathy, Fuwai Hospital, Chinese Academy of Medical Sciences and Peking Union Medical College, 167, Beilishilu, Xicheng District, Beijing, 100037 People’s Republic of China; 2 Department of Cardiology, Fuwai Hospital, National Center for Cardiovascular Diseases, Chinese Academy of Medical Sciences and Peking Union Medical College, 167, Beilishilu, Xicheng District, Beijing, 100037 People’s Republic of China; 3 State Key Laboratory of Cardiovascular Disease, Fuwai Hospital, National Center for Cardiovascular Diseases, Chinese Academy of Medical Sciences and Peking Union Medical College, 167, Beilishilu, Xicheng District, Beijing, 100037 People’s Republic of China

**Keywords:** Hypertension, TyG index, Cardiovascular risk, Young population

## Abstract

**Background:**

The triglyceride-glucose (TyG) index is a reliable marker of insulin resistance that is involved in the progression of hypertension. This study aimed to evaluate the association of the TyG index with the risk for major cardiovascular events (MACE) in young adult hypertension.

**Methods:**

A total of 2,651 hypertensive patients aged 18–40 years were consecutively enrolled in this study. The TyG index was calculated as Ln [triglycerides × fasting plasma glucose/2]. The cutoff value for an elevated TyG index was determined to be 8.43 by receiver-operating characteristic curve analysis. The primary endpoint was MACE, which was a composite of all-cause death, non-fatal myocardial infarction, coronary revascularization, non-fatal stroke, and end-stage renal dysfunction. The secondary endpoints were individual MACE components.

**Results:**

During the median follow-up time of 2.6 years, an elevated TyG index was associated with markedly increased risk of MACE (adjusted hazard ratio [HR] 3.440, *P* < 0.001) in young hypertensive adults. In subgroup analysis, the elevated TyG index predicted an even higher risk of MACE in women than men (adjusted HR 6.329 in women vs. adjusted HR 2.762 in men, P for interaction, 0.001); and in patients with grade 2 (adjusted HR 3.385) or grade 3 (adjusted HR 4.168) of hypertension than those with grade 1 (P for interaction, 0.024). Moreover, adding the elevated TyG index into a recalibrated Systematic COronary Risk Evaluation 2 model improved its ability to predict MACE.

**Conclusions:**

An elevated TyG index is associated with a higher risk of MACE in young adult hypertension, particularly in women and those with advanced hypertension. Regular evaluation of the TyG index facilitates the identification of high-risk patients.

**Graphical abstract:**

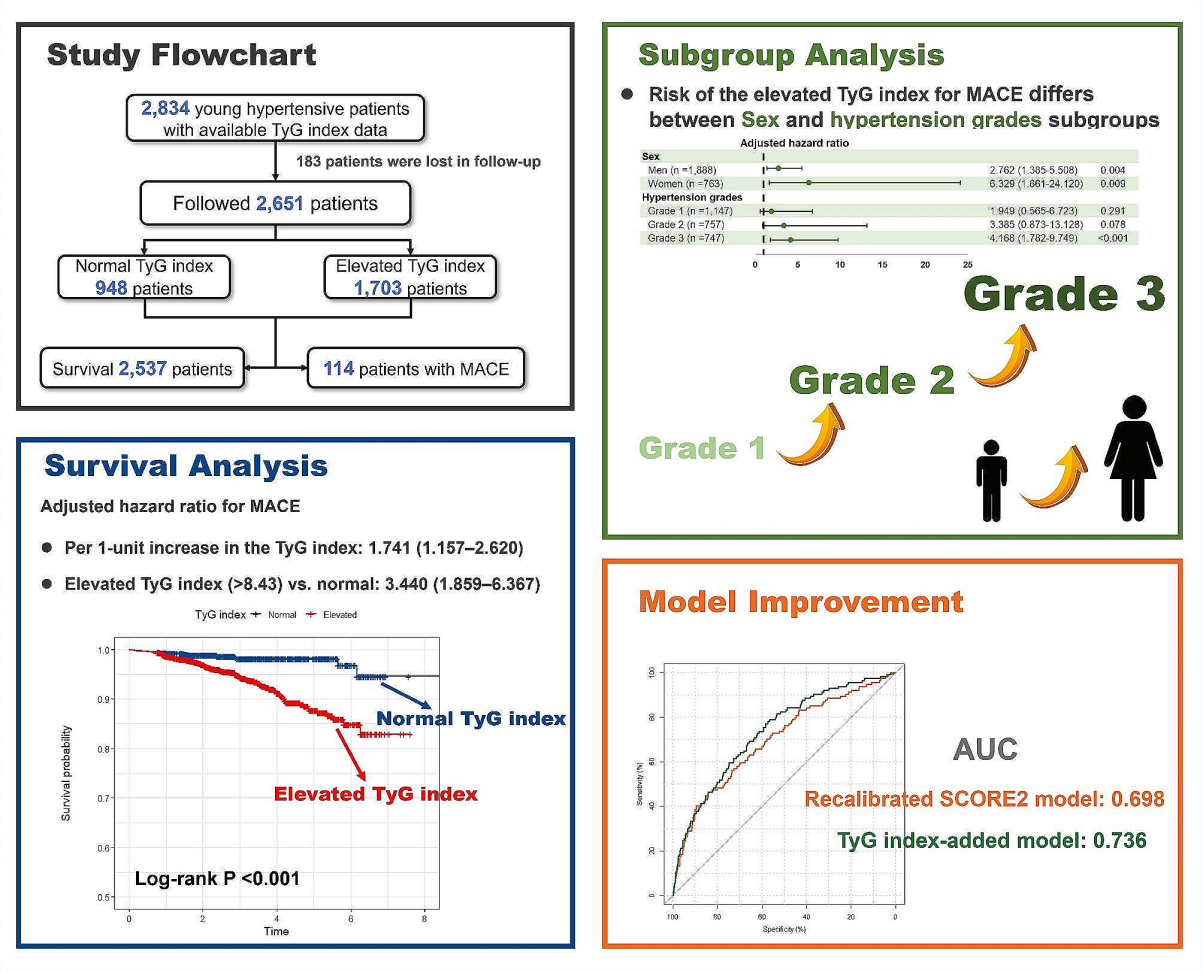

**Supplementary Information:**

The online version contains supplementary material available at 10.1186/s40885-024-00274-9.

## Introduction

Hypertension is a well-established risk factor for cardiovascular morbidity and mortality with a high prevalence of exposure [1]. The risks of hypertension-mediated organ damage and cardiovascular mortality are higher in young adults with hypertension than in normotensive subjects and elderly hypertensive patients [2, 3]. The results of a nationwide survey indicated that young people tend to overlook the adverse effects of hypertension, with much lower awareness, treatment, and control rates in comparison with their elderly counterparts [4]. Given that major adverse cardiovascular events (MACE) occur more often in patients older than 40 years of age, previous studies and guidelines have focused on the discrimination of hypertensive patients with cardiovascular risk in middle-aged or older populations [5]. Predictors that can be used to identify young hypertensive patients at high risk for short-term or long-term MACE are still unclear.

Insulin resistance is characterized by insulin insensitivity in peripheral tissues and shares pathological pathways in common with hypertension [6, 7]. In patients with hypertension, insulin resistance exacerbates endothelial dysfunction, arterial stiffness, and atherosclerosis, increasing the risks of organ damage and MACE [8–10]. Moreover, insulin resistance increases in prevalence across the decades from young adulthood onwards, and the predisposition to adverse cardiovascular outcomes becomes significant in later life [11–13]. 

The triglyceride-glucose (TyG) index is a novel and reliable surrogate marker of insulin resistance [14]. Previous studies have shown that an elevated TyG index is associated with higher incidences of subclinical atherosclerosis and cardiovascular mortality in middle-aged and elderly patients with hypertension [15–17]. However, the clinical significance of the TyG index in young adults with hypertension remains unclear.

In this study, we investigated the association between the TyG index and the risk of MACE in young hypertensive adults and analyzed its prognostic value in various subgroups. We also assessed whether the addition of the elevated TyG index into a recalibrated Systematic COronary Risk Evaluation 2 (SCORE2) score model would help to identify young adults with hypertension at high risk of MACE.

## Methods

### Study population

**Fig. 1:**
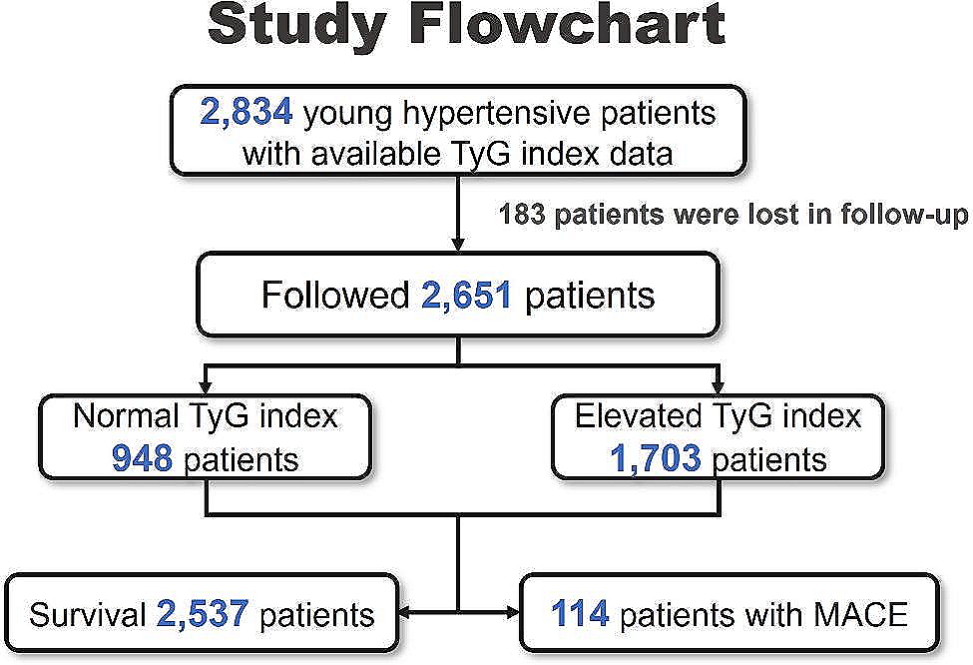
The flowchart of the study design. TyG index, triglyceride-glucose index; MACE, major adverse cardiovascular events

The study had a prospective observational design and enrolled 2,834 consecutively recruited patients aged 18–40 years old who were hospitalized with hypertension between 2012 and 2018 at Fuwai Hospital, Chinese Academy of Medical Science. All patients were available for the TyG index data, and a total of 2,651 patients were successfully followed and were finally included in our analyses (Fig. 1). Hypertension was defined as a systolic blood pressure (SBP) ≥ 140 mmHg and/or a diastolic blood pressure (DBP) ≥ 90 mmHg on three separate occasions in the clinic or regular use of antihypertensive medication. An SBP of 140–159 mmHg and/or a DBP of 90–99 mmHg was defined as grade 1 hypertension; an SBP of 160–179 mmHg and/or a DBP of 100–109 mmHg was defined as grade 2 hypertension; and an SBP ≥ 180 mmHg and/or a DBP ≥ 110 mmHg was defined as grade 3 hypertension.

This study protocol was approved by the Ethics Committee of Fuwai Hospital and conducted in accordance with the Declaration of Helsinki. Written informed consent was obtained from all study participants.

### Data collection

Blood pressure (BP) was measured as recommended after 15 min of relaxation by an oscillometric monitor (HP-1300; Omron, Fukuoka, Japan), and the average value of three measurements was recorded [18]. 

Blood samples were collected after a 12-hour overnight fast to measure triglyceride and fasting plasma glucose concentrations. The TyG index was calculated as Ln [triglycerides (mg/dl) × fasting plasma glucose (mg/dl)/2].

Left ventricular hypertrophy was identified by echocardiography as a left ventricular mass index (LVMI) > 95 g/m^2^ in women and > 115 g/m^2^ in men. Albuminuria was defined as a urinary microalbumin to creatinine ratio (UACR) of > 30 mg/g. Arterial stiffness was evaluated using a non-invasive limb BP monitor (BP-203RPEIII; Omron) as a brachial-ankle pulse wave velocity (baPWV) ≥ 14 m/s. Hypertensive retinopathy was assessed by the ophthalmologists and defined as fundus exudation, hemorrhage, or hypertensive retinopathy grade III or IV.

### Endpoint and follow-up

The outcome in each participant was determined by telephone interview or a clinical visit during follow-up. All events were carefully assessed and confirmed by trained investigators who were blinded to each patient’s baseline clinical characteristics. The primary endpoint of this study was MACE, which was defined as a composite of all-cause death, non-fatal myocardial infarction, coronary revascularization, non-fatal stroke, and end-stage renal dysfunction. The secondary endpoints were individual MACE components, which were all-cause death, coronary artery disease (CAD) events (fatal and non-fatal myocardial infarction, and coronary revascularization), stroke (fatal and non-fatal stroke), and renal events (death related to renal failure and end-stage renal dysfunction).

### Determination of the cutoff value for the TyG index

The receiver-operating characteristic curve was constructed to find the optimal cutoff of the TyG index for prediction of MACE. The C-statistic was 0.618 (95% CI 0.575–0.662), with the sensitivity of 0.868 and specificity of 0.369 (Additional file 1: Fig. S1). The optimal cutoff threshold value was 8.43 based on the maximal Youden index. In subsequent analyses, the cutoff value calculated by the receiver-operating characteristic curve was used to define patients with an elevated TyG index (> 8.43).

### Statistical analysis

Continuous variables are shown as the mean ± standard deviation or median [interquartile range] and categorical variables as the number (percentage). Baseline characteristics were compared between groups using the Student’s *t*-test or Mann-Whitney *U*-test if continuous and by the chi-squared test if categorical.

Survival curves were evaluated by the Kaplan–Meier method with the log-rank test. Univariable and multivariable Cox proportional hazards regression models were used to calculate the hazard ratio (HR) and 95% confidence interval (CI) and to evaluate the association between the TyG index and outcomes. The multivariable Cox model was adjusted for age, sex, body mass index (BMI), current smoking status, CAD, chronic renal disease, previous stroke, diabetes, hyperlipidemia, duration or the grades of hypertension, total cholesterol, low-density lipoprotein (LDL), high-density lipoprotein (HDL), and glycated hemoglobin. Interactions between the TyG index and sex or the grades of hypertension were estimated in the above-mentioned multivariable model. The Pearson correlation test was used to assess the relationship between the TyG index and the LVMI, UACR, and baPWV.

The risk model was recalibrated to our data on the basis of variables recommended in the SCORE2 model, including age, sex, current smoking status, SBP, total cholesterol, and HDL, to predict the risk of MACE. The incremental predictive value of the elevated TyG index beyond the recalibrated SCORE2 model was assessed by the C-statistic, integrated discrimination improvement, and continuous net reclassification improvement. The statistical analysis was performed using R version 4.2.3 (R Core Team, Vienna, Austria). A two-sided *P*-value < 0.05 was considered statistically significant.

## Results

The baseline characteristics of the study participants are summarized according to whether their TyG index was normal or elevated in Table 1. The mean patient age was 31.9 ± 5.8 years and 71.2% were male. Compare to those with normal TyG index, patients with an elevated TyG index were older, more likely to be men, were more likely to have a higher BMI, a higher grade of hypertension, and a higher prevalence of current smoking, CAD, chronic renal disease, diabetes, and hyperlipidemia. In terms of hypertension-mediated organ damage, albuminuria and arterial stiffness were more common in patients with the elevated TyG index. Further analyses revealed significant positive correlations of the TyG index with LVMI (*r* = 0.088, *P* < 0.001), UACR (*r* = 0.145, *P* < 0.001), and baPWV (*r* = 0.162, *P* < 0.001) (Additional file 2: Fig. S2).

**Table 1:** Baseline characteristics of the study population

Characteristics	Total	Patients with a normal TyG index	Patients with an elevated TyG index	*P* value
	*N = 2,651*	*N = 948*	*N = 1,703*	
Age, yrs	31.9 ± 5.8	30.1 ± 6.5	32.9 ± 5.2	< 0.001
Men, n, (%)	1888 (71.2)	524 (55.3)	1364 (80.1)	< 0.001
BMI, g/m^2^	26.8 ± 4.6	24.6 ± 4.4	28.1 ± 4.2	< 0.001
Hypertension duration, yrs	4.2 ± 4.3	3.84 ± 4.1	4.34 ± 4.5	0.004
Current smoking status, n, (%)	976 (36.8)	209 (22.0)	767 (45.0)	< 0.001
CAD, n, (%)	78 (2.9)	14 (1.5)	64 (3.8)	0.001
Chronic renal disease, n, (%)	87 (3.3)	16 (1.7)	71 (4.2)	< 0.001
Chronic renal failure, n, (%)	6 (0.2)	0 (0.0)	6 (0.4)	0.095
Previous Stroke, n, (%)	106 (4.0)	34 (3.6)	72 (4.2)	0.470
Diabetes, n, (%)	200 (7.5)	17 (1.8)	183 (10.7)	< 0.001
Hyperlipidemia, n, (%)	929 (35.0)	119 (12.6)	810 (47.6)	< 0.001
Heart rate, beats/min	78.5 ± 12.8	77.7 ± 12.8	79.0 ± 12.8	0.009
Office SBP, mmHg	154.6 ± 21.0	154.1 ± 20.3	154.8 ± 21.4	0.387
Office DBP, mmHg	99.1 ± 16.2	97.7 ± 16.2	99.8 ± 16.2	0.002
Hypertension Grades				
Grade 1, n, (%)	1,147 (43.3)	424 (44.7)	723 (42.5)	0.496
Grade 2, n, (%)	757 (28.6)	267 (28.2)	490 (28.8)	
Grade 3, n, (%)	747 (28.2)	257 (27.1)	490 (28.8)	
Triglyceride, mmol/l	1.82 ± 1.31	0.88 ± 0.23	2.34 ± 1.37	< 0.001
Total cholesterol, mmol/l	4.61 ± 0.99	4.22 ± 0.83	4.82 ± 1.00	< 0.001
LDL, mmol/l	2.87 ± 0.83	2.59 ± 0.71	3.03 ± 0.84	< 0.001
HDL, mmol/l	1.18 ± 0.35	1.36 ± 0.36	1.08 ± 0.31	< 0.001
Fast plasma glucose, mmol/l	5.03 ± 1.17	4.62 ± 0.59	5.26 ± 1.34	< 0.001
HbA1c, %	5.48 ± 0.66	5.26 ± 0.36	5.60 ± 0.76	< 0.001
LVMI, g/m^2^	94.7 ± 30.2	91.9 ± 30.1	96.3 ± 30.1	< 0.001
Left ventricular hypertrophy, n, (%)	583 (22.0)	210 (22.2)	373 (21.9)	0.883
UACR, mg/g	26.4 [14.4–54.6]	22.6 [12.8–40.5]	28.6 [15.5–67.3]	< 0.001
Albuminuria, n, (%)	1,449 (43.3)	348 (36.7)	801 (47.0)	< 0.001
baPWV, m/s	14.7 ± 2.7	14.2 ± 2.5	14.9 ± 2.7	< 0.001
Arterial stiffness, n, (%)	1,458 (55.0)	457 (48.2)	1,001 (58.8)	< 0.001
Hypertensive retinopathy, n, (%)	103 (3.9)	28 (3.0)	75 (4.4)	0.074

TyG index: triglyceride-glucose index; BMI: body mass index; CAD: coronary artery disease; SBP: systolic blood pressure; DBP; diastolic blood pressure; LDL: low-density lipoprotein; HDL: high-density lipoprotein; HbA1c: glycerate hemoglobin; LVMI: left ventricular mass index; UACR: urinary microalbumin to creatinine ratio; baPWV: brachial-ankle pulse wave velocity

**Fig. 2:**
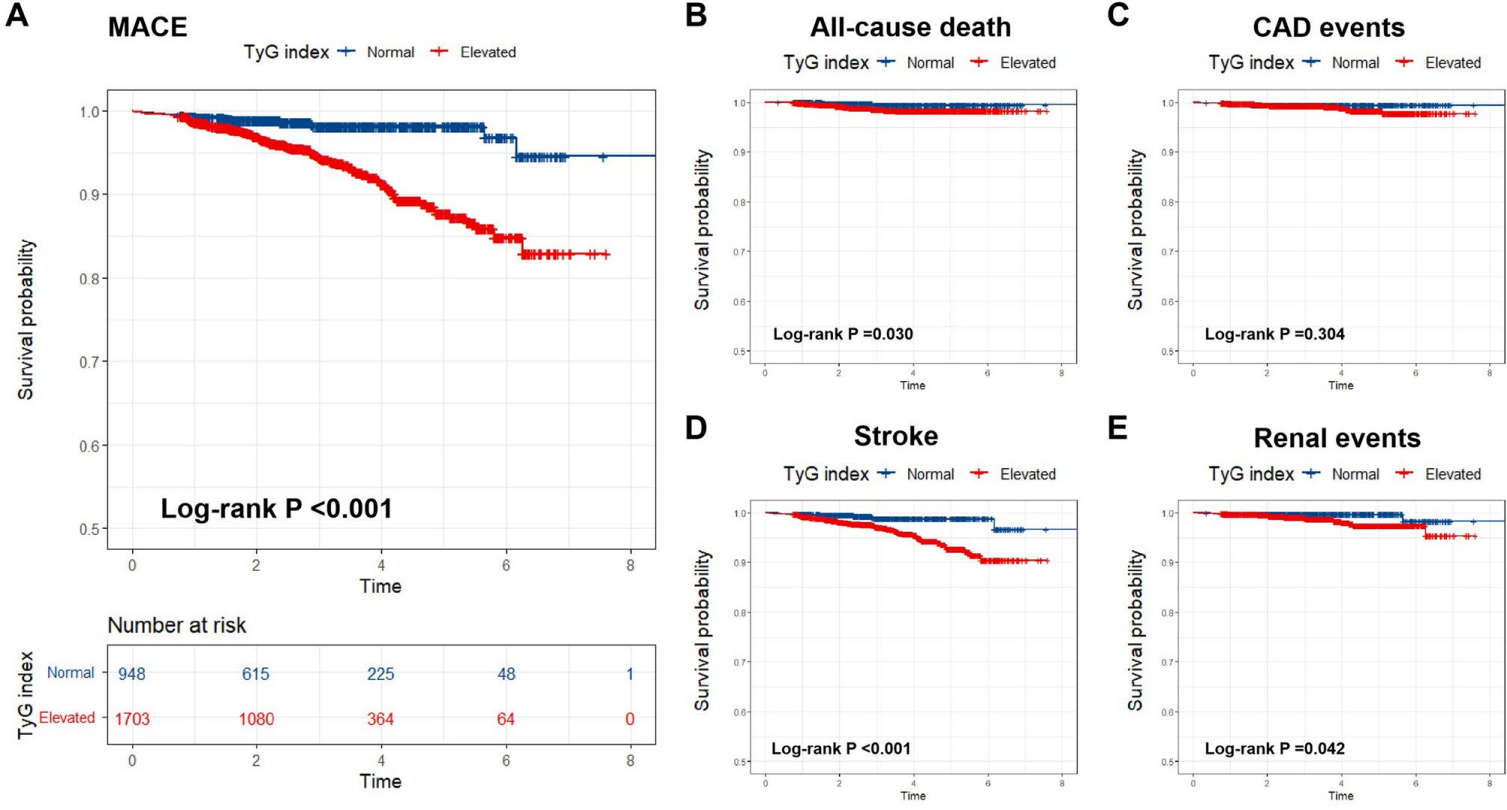
Kaplan-Meier survival curves for (**A**) MACE and (**B-E**) individual MACE components by the TyG index. MACE, major adverse cardiovascular events; TyG index, triglyceride-glucose index; CAD, coronary artery disease

During a median follow-up of 2.6 years, 114 (4.3%) of the 2,651 study participants experienced MACE. The individual MACE components were summarized in the Additional file 3: Table S1. Both univariable and multivariable models evaluating the TyG index as a continuous variable showed that a higher TyG index was associated with an increased risk of MACE (adjusted HR 1.741, 95% CI 1.157–2.620, *P* < 0.001; Table 2). Meanwhile, a higher TyG index was also shown to be associated with an increased risk of stroke (adjusted HR 1.763, 95% CI 1.024–3.033, *P* = 0.041; Table 2). When dichotomized the TyG index level, the Kaplan–Meier curves showed a higher incidence of MACE (*P* < 0.001), all-cause death (*P* = 0.030), stroke (*P* < 0.001) and renal events (*P* = 0.042) in patients with an elevated TyG index during follow-up (Fig. 2). Both the univariable and multivariable models showed that an elevated TyG index (> 8.43) was associated with a significantly higher risk of MACE (adjusted HR 3.440, 95% CI 1.859–6.367, *P* < 0.001) and stroke (adjusted HR 3.135, 95% CI 1.366–7.193, *P* =0.007) in young patients with hypertension (Table 2).

**Table 2:** Associations between an elevated TyG index and outcomes

Outcomes	Unadjusted HR (95% CI)	Unadjusted *P*-value	Adjusted HR (95% CI)	Adjusted *P*-value
**MACE**				
TyG index (per 1-unit increase)	1.745 (1.340–2.273)	< 0.001	1.741 (1.157–2.620)	0.008
Elevated TyG index (cut-off value of 8.43)^*^	3.854 (2.239–6.636)	< 0.001	3.440 (1.859–6.367)	< 0.001
**Individual MACE components**				
**All-cause death**				
TyG index (per 1-unit increase)	1.193 (0.637–2.237)	0.582	1.365 (0.512–3.640)	0.535
Elevated TyG index (cut-off value of 8.43)^*^	3.537 (1.047–11.952)	< 0.001	3.689 (0.961–14.160)	0.057
**CAD events**				
TyG index (per 1-unit increase)	1.645 (0.872–3.104)	0.125	1.364 (0.484–3.843)	0.557
Elevated TyG index (cut-off value of 8.43)^*^	1.690 (0.614–4.650)	0.310	1.159 (0.322–4.174)	0.821
**Stroke**				
TyG index (per 1-unit increase)	1.867 (1.311–2.658)	< 0.001	1.763 (1.024–3.033)	0.041
Elevated TyG index (cut-off value of 8.43)^*^	3.963 (1.888–8.322)	< 0.001	3.135 (1.366–7.193)	0.007
**Renal events**				
TyG index (per 1-unit increase)	1.572 (0.878–2.814)	0.128	1.742 (0.671–4.524)	0.255
Elevated TyG index (cut-off value of 8.43)^*^	2.902 (0.991–8.496)	0.052	2.103 (0.718–6.163)	0.175

TyG index, triglyceride-glucose index; MACE, major cardiovascular events; HR, hazard ratio; CI, confidence interval; CAD, coronary artery disease

The multivariable Cox proportional regression models were adjusted for age, sex, body mass index, hypertension duration, hypertension grades, current smoking status, CAD, chronic renal disease, previous stroke, diabetes, hyperlipidemia, total cholesterol, low-density lipoprotein, high-density lipoprotein, and glycated hemoglobin

* Patients with a normal TyG index were used as reference

Interaction tests and analyses stratified by sex and the grades of hypertension were performed (Table 3). An elevated TyG index was associated with a higher risk of MACE regardless of sex; however, this risk was more pronounced in women than men (adjusted HR 2.762, 95% CI 1.385–5.508, *P* = 0.004 in men; adjusted HR 6.329, 95% CI 1.661–24.120, *P* = 0.009 in women; P for interaction, < 0.001). Notably, 1,147 patients (43.3%) had grade 1 hypertension, 757 (28.6%) had grade 2, and 747 (28.2%) had grade 3. The adjusted HR increased progressively from grade 1 to grade 3 hypertension, suggesting that the predictive value was more marked in the advanced grades of hypertension (adjusted HR 1.949, 95% CI 0.565–6.723, *P* = 0.291 for grade 1; adjusted HR 3.385, 95% CI 0.873–13.128, *P* = 0.078 for grade 2; and adjusted HR 4.168, 95% CI 1.782–9.749, *P* < 0.001 for grade 3; P for interaction, 0.024).

**Table 3:** Stratification analysis of risk of an elevated TyG index for MACE

Subgroups	Unadjusted HR* (95% CI)	Unadjusted *P*-value	Adjusted HR* (95% CI)	Adjusted *P*-value	*P* for interaction
**Sex subgroups**					
Men (*n* = 1,888)	2.830 (1.507–5.312)	0.001	2.762 (1.385–5.508)	0.004	< 0.001
Women (*n* = 763)	5.990 (2.025–17.713)	0.001	6.329 (1.661–24.120)	0.009	
**Hypertension grades subgroups**					
Grade 1 (*n* = 1,147)	2.937 (1.008–8.558)	0.048	1.949 (0.565–6.723)	0.291	0.024
Grade 2 (*n* = 757)	4.636 (1.396–15.399)	0.012	3.385 (0.873–13.128)	0.078	
Grade 3 (*n* = 747)	4.034 (1.920–8.493)	< 0.001	4.168 (1.782–9.749)	< 0.001	

TyG index, triglyceride-glucose index; MACE, major cardiovascular events; HR, hazard ratio; CI, confidence interval

The multivariable Cox proportional regression models were adjusted for age, sex, body mass index, hypertension duration, hypertension grades, current smoking status, CAD, chronic renal disease, previous stroke, diabetes, hyperlipidemia, total cholesterol, low-density lipoprotein, high-density lipoprotein, and glycated hemoglobin

* Patients with a normal TyG index were used as reference

**Fig. 3:**
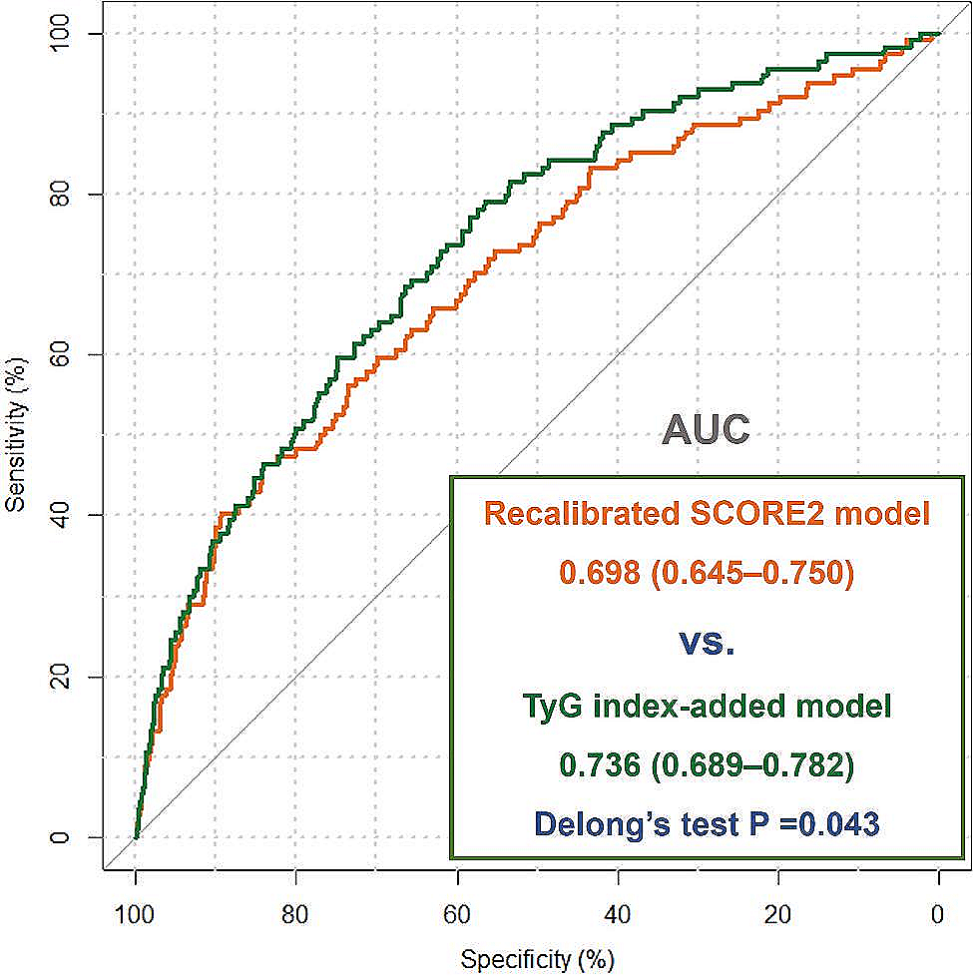
The improvement of adding the elevated TyG index into the recalibrated SCORE2 model for predicting MACE in patients with young adult hypertension. TyG index, triglyceride-glucose index; SCORE2, Systematic COronary Risk Evaluation 2; MACE, major adverse cardiovascular events

Finally, we assessed whether addition of the elevated TyG index had incremental predictive value over traditional risk factors for hypertension in young adults. When the elevated TyG index was added into the recalibrated SCORE2 model, a significant improvement was observed in discrimination and reclassification for predicting MACE (C-statistics: for risk model + TyG index vs. risk model alone, 0.736, 95% CI 0.689–0.782 vs. 0.698, 95% CI 0.645–0.750, *P* = 0.043; integrated discrimination improvement, 0.008, 95% CI 0.001–0.015, *P* = 0.020; continuous net reclassification improvement, 0.365, 95% CI 0.195–0.505, *P* < 0.001; Fig. 3).

## Discussion

This study is the first to demonstrate a strong association between an elevated TyG index and increased risks of MACE and stroke in young adult hypertension. The elevated TyG index-associated risk was particularly high in young women and patients with advanced grades of hypertension. Furthermore, our results also demonstrate that adding the elevated TyG index into the recalibrated SCORE2 model provides incremental value on its discrimination and reclassification ability to estimate the risk of MACE.

Insulin is a vascular hormone with key roles in metabolic and hemodynamic hemostasis [19, 20]. Insulin resistance induces hyperinsulinemia and hyperglycemia in hypertensive subjects, activating oxidative stress and inflammation and upregulating the activity of the renin-angiotensin-aldosterone-system [8, 13, 21]. Both elevated BP and insulin resistance result in endothelial dysfunction, promoting atherosclerosis and target organ damage [10, 20, 22]. An elevated TyG index, which is a convenient marker of insulin resistance, is positively associated with higher incidences of albuminuria and arterial stiffness in middle-aged and elderly patients with hypertension [16, 23, 24]. A baseline or persistently higher TyG index during puberty is also associated with a higher incidence of arterial stiffness in adulthood. Moreover, an elevated TyG index has been found to be a strong risk factor for renal dysfunction in both the general young adult population and the young population with diabetes [25, 26]. In line with these reports, our present study found that an elevated TyG index was associated with higher UACR and baPWV values in young adults with hypertension, resulting in a higher prevalence of albuminuria and arterial stiffness. This finding adds to the robust clinical evidence that elevated TyG index is related to subclinical atherosclerosis. A previous study identified an association between a higher TyG index and a higher LVMI in elderly patients with hypertension [16], and our present findings affirm that LVMI also increased with the TyG index in hypertensive adults at a young age. However, the prevalence of left ventricular hypertrophy presented was comparable between patient with or without elevated TyG index. Considering that the development of left ventricular hypertrophy requires a prolonged period of time with hypertension [27, 28], it is plausible to suggest that patients with elevated TyG index in our study might be too young to progress from increased LVMI to left ventricular hypertrophy; yet they may experience a high prevalence of left ventricular hypertrophy later in life.

Previous studies have demonstrated that an elevated TyG index is predominantly associated with a higher risk of adverse cardiovascular events in the middle-aged and elderly population with hypertension [4, 15, 17, 29]. Consistent with the earlier reports, our present study confirms for the first time that an elevated TyG index predicts a significantly increased risk of MACE and stroke in young adults with hypertension. A sex-related difference in the interaction between the TyG index and adverse cardiovascular outcomes has already been reported, in that an elevated TyG index was found to be a stronger predictor of myocardial infarction in women than in men in the general population [30]. Our study similarly found that the ability of an elevated TyG index to predict MACE was particularly high in young women with hypertension. However, another study did not find a significant sex-related difference in the ability of the TyG index to predict MACE in an older population [15]. These inconsistent findings might reflect the fact that menopausal and older women have reduced estrogen levels [31], abolishing the protective effects of women and diminishing the sex differences. In young women, estrogen could alleviate insulin resistance to some extent and the TyG index would be relatively lower. Therefore, when the TyG index is elevated to the same cutoff value as in men, the predictive efficacy is greater in women than in men.

In general, the grade of hypertension is an important consideration when evaluating the risk of a poor prognosis in patients with hypertension, and those in the advanced grades of hypertension often need specific attention and risk stratification [18]. Of note, we found that the likelihood of an elevated TyG index increased with increasing grade of hypertension. One possible explanation for this observation is that insulin resistance promotes elevation of BP and vice versa. For example, it has been demonstrated in animal models that insulin resistance promotes BP elevation by impairing synthesis of nitric oxide, while elevated BP impairs glucose intake by altering the delivery of insulin and glucose to skeletal muscle cells [32]. Therefore, in patients with hypertension, there is a positive relationship between the severity of insulin resistance and the grade of hypertension [33]. 

The literature indicates that the global burden of cardiovascular disease has plateaued in the middle-aged and elderly population but is still increasing in the young population [34]. Although several risk stratification models have been designed to identify high-risk patients with hypertension aged older than 40 years, there is no widely recognized model that can predict cardiovascular risk in young individuals with hypertension [5, 35]. The SCORE2 model is recommended in the current guideline for evaluation of the long-term risk of fatal and non-fatal cardiovascular events in middle-aged and elderly patients with hypertension. However, our study population was much younger with a short follow-up period. Therefore, we assume that it was reasonable to recalibrate the SCORE2 model based on our data in order to evaluate the potential cardiovascular risk in the young population with hypertension. In our study, the ability to identify patients at a high risk of MACE was significantly improved when the elevated TyG index was added into the recalibrated SCORE2 model. The incremental impact of the elevated TyG index beyond the established risk model has also been shown in elderly patients with cardiovascular disease [36]. Considering the cost-effectiveness and ready clinical availability of the TyG index, we support its use as an efficient marker for stratification of cardiovascular risk in young adults with hypertension.

This study has some limitations. First, only a small proportion of our study participants had diabetes, which is more common in the elderly. Therefore, we could not perform a subgroup analysis based on diabetes status. Given that insulin resistance is closely associated with diabetes and the prognostic value of the TyG index might be affected by diabetes status, we included it as a covariable in our multivariable Cox model and found that the increased risk of MACE in patients with an elevated TyG index was independent of diabetes status. Similar results have been reported in elderly hypertensive patients without diabetes, demonstrating that an elevated TyG index is independently associated with a higher risk of MACE ^19,40^. Second, our study population consisted of patients admitted to a single tertiary institution, which may limit the generalizability of our findings.

## Conclusions

An elevated TyG index is a strong predictor of a poor prognosis in young adults with hypertension, especially in women and those with advanced hypertension. Adding the elevated TyG index into the SCORE2 model has incremental value to identify patients at high risk to MACE in young adult hypertension. This study filled the gap of prognostic predictors and improved the identification of patients with high cardiovascular risk in young adult hypertension.

### Electronic supplementary material

Below is the link to the electronic supplementary material.

**Supplementary Material 1**: Fig. S1. The receive-operating characteristic curve of the TyG index and MACE

**Supplementary Material 2**: Fig. S2. The associations between the TyG index and the parameters of hypertension-mediated organ damage

**Supplementary Material 3**: Table S1. Individual MACE components during follow-up period

## Data Availability

The datasets used/or analyzed during the current study are available from the corresponding author on reasonable request.

## Abbreviations

BaPWV: brachial-ankle pulse wave velocity
BMI: body mass index
CAD: coronary artery disease
CI: confidence interval
DBP: diastolic blood pressure
HDL: high-density lipoprotein
HR: hazard ratio
LDL: low-density lipoprotein
LVMI: left ventricular mass index
MACE: major cardiovascular events
SBP: systolic blood pressure
SCORE2: Systematic COronary Risk Evaluation 2
TyG index: triglyceride-glucose index
UACR: urinary microalbumin to creatinine ratio
