# Supplementary figures and images for: Prognostic value of the triglyceride-glucose index for adverse cardiovascular outcomes in young adult hypertension
Source: Clin Hypertens. 2024 Sep 1;30:25. doi: 10.1186/s40885-024-00274-9 (PMC11366158; doi:10.1186/s40885-024-00274-9)

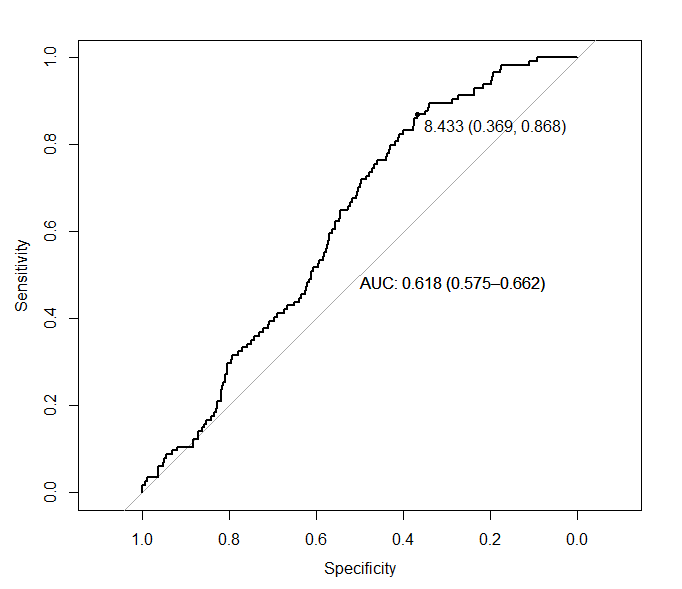

Supplement: Supplementary file 1 — Supplementary Material 1: Fig. S1. The receive-operating characteristic curve of the TyG index and MACE [file 40885_2024_274_MOESM1_ESM.png]

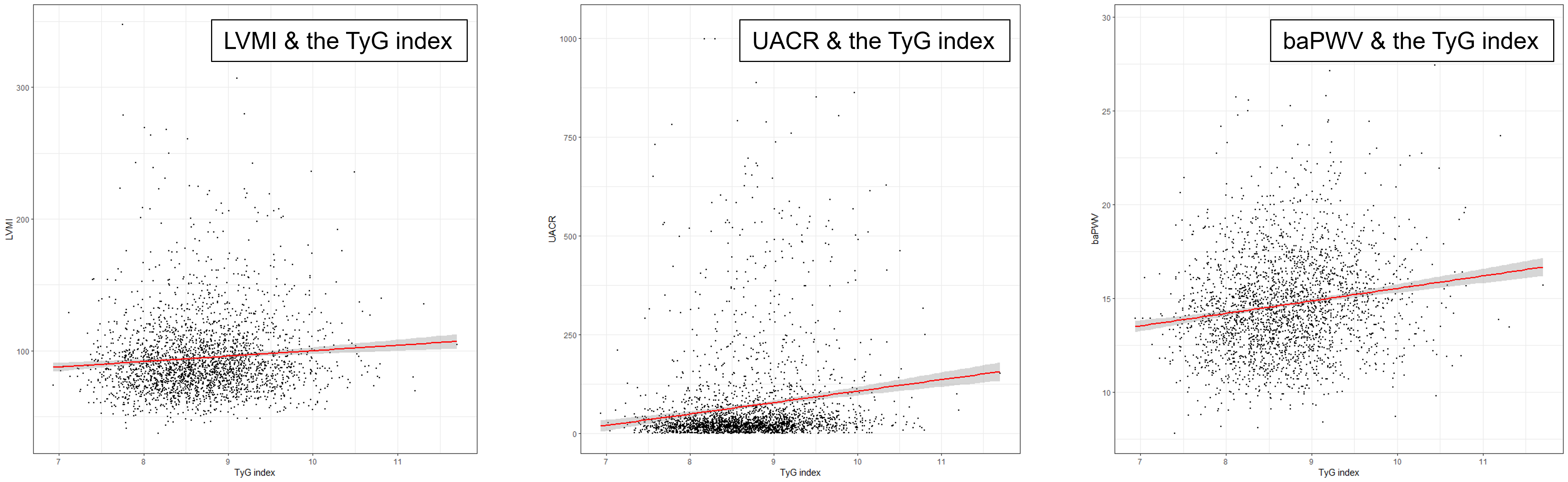

Supplement: Supplementary file 2 — Supplementary Material 2: Fig. S2. The associations between the TyG index and the parameters of hypertension-mediated organ damage [file 40885_2024_274_MOESM2_ESM.png]
